# The N-terminus of *Paenibacillus larvae* C3larvinA modulates catalytic efficiency

**DOI:** 10.1042/BSR20203727

**Published:** 2021-01-06

**Authors:** Madison Turner, Kayla A. Heney, A. Rod Merrill

**Affiliations:** 1 Department of Molecular and Cellular Biology, University of Guelph, Guelph, Ontario N1G 2W1, Canada; 2 Department of Biochemistry, McGill University, Montreal, Quebec H3G 0B1, Canada

**Keywords:** ADP-ribosyltransferase toxins, enzyme mechanisms, honey bee diseases, macrophage cell entry, protein-protein interactions

## Abstract

C3larvinA was recently described as a mono-ADP-ribosyltransferase (mART) toxin from the enterobacterial repetitive intergenic consensus (ERIC) III genotype of the agricultural pathogen, *Paenibacillus larvae*. It was shown to be the full-length, functional version of the previously described C3larvin_trunc_ toxin, due to a 33-residue extension of the N-terminus of the protein. In the present study, a series of deletions and substitutions were made to the N-terminus of C3larvinA to assess the contribution of the α_1_-helix to toxin structure and function. Catalytic characterization of these variants identified Asp^23^ and Ala^31^ residues as supportive to enzymatic function. A third residue, Lys^36^, was also found to contribute to the catalytic activity of the enzyme. Analysis of the C3larvinA homology model revealed that these three residues were participating in a series of interactions to properly orient both the Q-X-E and S-T-S motifs. Ala^31^ and Lys^36^ were found to associate with a structural network of residues previously identified *in silico*, whereas Asp^23^ forms novel interactions not previously described. At last, the membrane translocation activity into host target cells of each variant was assessed, highlighting a possible relationship between protein dipole and target cell entry.

## Introduction

Mono-ADP-ribosyltransferase (mART) toxins are an exoenzyme class used by pathogenic bacteria to confer damage to host cells [1–9]. These virulence factors function by binding an NAD^+^ molecule and catalyzing the transfer of the ADP-ribose moiety on to a target macromolecule [10–13]. Covalent modification of these macromolecules, such as G_αs_, RhoA and DNA, results in altered cellular function and can lead to cell death [14–19]. This enzyme class can also exhibit glycohydrolase (GH) activity in the absence of a target macromolecule, where the glycosidic bond of NAD^+^ is hydrolyzed to release ADP-ribose and nicotinamide [20–23]. Human pathogens, including *Vibrio cholera, Clostridium botulinum* and *Corynebacterium diphtheriae* have been previously described to use mART toxins [4,24–27]; however, more recently mART toxins were discovered in the agricultural pathogen, *Paenibacillus larvae* [28–32].

*P. larvae* is a Gram-positive, spore-forming bacterium, and the causative agent of American Foulbrood (AFB) disease [33–36]. This lethal infection targets honeybee larvae, and has led to the loss of hives worldwide through the destruction of colony progeny [33,34,37]. AFB is transmitted through bacterial spores, which are inadvertently fed to naïve larvae by nurse bees [34,38]. The spores quickly propagate within the larval midgut, and eventually the bacteria begin to attack the cell–cell and cell–matrix junctions of the midgut epithelial lining [33,38]. Breaching this barrier results with access to the hemocoel, or main body cavity of the host, where the bacteria will feed on larval tissues. As nutrient sources are depleted, *P. larvae* sporulates, re-forming the infectious agent of the disease. Host death correlates to the breach of the epithelial lining, which can take 7–12 days depending on the *P. larvae* genotype present in the infection [38].

Four genotypes of *P. larvae* have been established using by mass spectrometry [39] and repetitive-element PCR analysis [40,41]. Primers specific to enterobacterial repetitive intergenic consensus (ERIC) sequences revealed four distinct banding patterns, resulting in the nomenclature of *P. larvae* ERIC I–IV. The genotypes differ greatly from one another, including colony phenotype, energy metabolism and toxin production [33,40]. This also applies to the associated mART toxins expressed by each genotype. To date, three mART toxins have been characterized in *P. larvae*: Plx2A in ERIC I, C3larvin_trunc_ in ERIC I and II, and C3larvinA in ERIC III [28,29,32].

The first mART toxin to be enzymatically characterized from *P. larvae* was C3larvin_trunc_, previously denoted as C3larvin [32]. It was shown to be a single-domain toxin that targeted RhoA through a catalytic Q-X-E motif, resulting in its classification as a C3-like mART toxin. However, unlike other C3 toxins, C3larvin_trunc_ failed to intoxicate target macrophage cells despite being lethal when expressed in yeast cells. It was determined that the protein had a truncated N-terminal α_1_-helix which led to an inability to gain entry to host cells and cause intracellular damage [32]. The full-length protein, C3larvinA, was later identified in the ERIC III genotype [29,30]. Like C3larvin_trunc_, C3larvinA targets RhoA for ADP-ribosylation through a catalytic Q-X-E motif, and possesses GH activity; however, the rate of reaction was 12-fold, and 200-fold higher than that of C3larvin_trunc_, respectively. Additionally, C3larvinA has the necessary N-terminal machinery (α-helix 1) to translocate the host cell membrane and initiate infection [29]. These findings further confirm the importance of the N-terminus in cell entry as well as enzymatic activity.

In the present study, the role of the N-terminus is examined in relation to the structure, enzyme activity and cell entry of C3larvinA. The characterization of N-terminal variants showed decreased protein stability and enzymatic activity in relation to the wild-type (WT) toxin. These findings support the importance of residues previously identified through computational studies, while further identifying novel interactions between the α_1_-helix and the ADP-ribosyl-turn-turn (ARTT)-loop, which houses the catalytic Q-X-E motif [42]. At last, C3larvinA was shown to have improved RhoA-targeting capabilities within macrophage cells compared with C3larvin_trunc_. However, these results indicated that the differences were not attributed to the 33-residue extension at the N-terminus of the protein.

## Materials and methods

### Protein expression and purification

All C3larvinA WT and variant proteins were expressed and purified as previously described [29]. Briefly, protein expression was induced in *Escherichia coli* BL21 λDE3 cells using 1 mM isopropyl β-d-1-thiogalactopyranoside (IPTG) at 37°C for 4 h. Cells were harvested through centrifugation and resuspended in 500 mM NaCl and 50 mM Tris/HCl, pH 7.5. An Emulsiflex C3 high-pressure homogenizer (Avestin Inc., Ottawa, Canada) was used to lyse the cells in the presence of 120 µM PMSF, 50 µg/ml CHAPS, 100 µg/ml DNase and 1 mM EDTA before a second round of centrifugation. After incubation with 10 mM MgCl_2_, the protein of interest was purified from the soluble fraction using a combination of metal-affinity and size-exclusion chromatography.

### Differential-scanning fluorimetry

The thermal stability of each variant was assessed using the Protein Thermal Shift dye, SYPRO Orange™ (Invitrogen, Massachusetts, U.S.A.), while fluorescence was monitored with a StepOnePlus Real-Time PCR system (Applied Biosystems, Foster City, U.S.A.). Experiments were carried out in triplicate in 500 mM NaCl and 10 mM Tris/HCl, pH 7.5 with a final protein concentration of 0.5 mg/ml and dye concentration of 1×.

### STRUM analysis

The relative fold change in stability of each single-residue variant compared with C3larvinA WT was assessed using the STRUM server [43]. This method uses multiple-sequence alignments, threading template alignments and i-TASSER structure prediction models to predict the fold stability change caused by single-residue changes to the protein sequence [43]. The C3larvinA homology model was submitted to the STRUM server, which then reported the fold stability change (ΔΔG), for each substitution. A fold change below zero indicates that the mutation is destabilizing to protein structure, while a score above zero indicates the mutation is stabilizing the protein structure.

### NAD^+^-binding

The affinity of each variant for the NAD^+^ substrate was assessed through a tryptophan-quenching assay [44]. A buffer solution (50 mM NaCl, 20 mM Tris/HCl, pH 7.9) at an initial volume of 600 µl containing 1.25 µM protein was titrated with β-nicotinamide adenine dinucleotide (β-NAD^+^) to achieve a range of substrate concentrations between 1 and 1000 µM. Measurements were taken using a Cary Eclipse fluorescence spectrophotometer (Varian Instruments, Mississauga, Canada) with an excitation wavelength of 295 nm, emission wavelength of 340 nm and excitation and emission bandpasses of 5 nm. Measurements were taken in triplicate using 0.5 mm × 0.5 mm fluorescence quartz cuvettes. Kinetic values were calculated on GraphPad Prism ver 5.0 (San Diego, U.S.A.).

### GH activity

The GH activity of each protein was assessed using etheno-adenosine monophosphate (ε-NAD^+^) as the substrate [21,29]. The reaction was held at 25°C in a reaction buffer of 50 mM NaCl and 20 mM Tris/HCl, pH 7.9. Measurements were made at a protein concentration of 20 µM and ε-NAD^+^ concentrations ranging from 0 to 500 µM, using an excitation wavelength of 305 nm, emission wavelength of 405 nm and an excitation and emission bandpass of 5 nm. The reaction was monitored for 5 min, and the resulting slope was converted from fluorescence units into product concentration using a standard ε-AMP curve. All measurements were carried out in triplicate and kinetic values were calculated using GraphPad Prism ver. 5.0 (San Diego, CA).

### C3larvinA homology model

The C3larvinA homology model was built as described earlier [29]. Briefly, the 1.65 Å crystal structure of Plx2A (PDB: 5URP; 55% sequence identity) was used as a template to model the C3larvinA structure in Phyre2 [45]. The resulting homology model was reported with 100% confidence, and shared similar topology to other C3-toxins [29].

### Cell morphology assay

J774A.1 mouse macrophage cells were maintained in Dulbecco’s modified Eagle’s medium (DMEM) with 10% fetal bovine serum (FBS), 100 U/ml penicillin and 100 µg/ml streptomycin as previously described [32]. Cells were grown at 37°C in a humidified 5% CO_2_ incubator. Cells were lifted by scraping and diluting ten-fold in complete growth medium. To assess the effect of each protein on cell morphology, confluent cells were diluted to 250000 cells/ml and mixed with 300 nM of toxin. From this cell suspension, 37500 cells were seeded in triplicate in a 96-well plate and incubated for 20 h. Cells were then assessed and imaged under a Nikon TMS inverted phase-contrast microscope with the 20× objective (Nikon Canada; Mississauga, Canada) to identify any morphological changes.

### Fluorescence microscopy

Purified protein in 0.5 M NaCl, 0.1 M Na_3_PO_4_, pH 7.5 was conjugated with Dylight 488 NHS Ester (Thermo Fisher Scientific, Massachusetts, U.S.A.) according to manufacturer’s instructions, with the following exception: a two- to three-fold molar excess of dye was used in place of the recommended eight- to ten-fold to maintain a molar ratio of labeled protein to dye under 2.0. Excess dye was then removed through dialysis coupled with buffer exchange to yield conjugated protein in a final buffer system of 500 mM NaCl, 50 mM Tris/HCl, pH 7.5.

J774A.1 mouse macrophage cells were prepared as described above. Cells were seeded at 500000 cells per well in a six-well plate prepared with sterile glass coverslips and incubated overnight at 37°C with 5% CO_2_. Cells were then treated with 300 nM of toxin–Dylight 488 conjugate and incubated for 4 h. All media were removed after the incubation step and cells were washed three times with PBS. Note that all following wash steps were performed in triplicate with prewarmed PBS. Cells were fixed with buffered 4% paraformaldehyde (PFA) solution for 15 min on ice. Cells were then washed, incubated with 150 mM glycine in PBS for 15 min at room temperature, and washed again. Cells were permeabilized with 0.5% Triton X-100 in PBS for 10 min at room temperature. After washing, the cells were treated with 2 µg/ml of 2-(4-amidinophenyl)-1H-indole-6-carboxamidin (DAPI) in methanol for 15 min at room temperature, followed by a final wash. During co-localization studies, additional steps were carried out to target cellular RhoA before the final incubation with DAPI. Following permeabilization, samples were blocked with 5% BSA in PBS for 1.5 h after permeabilization with Triton X-100. Cells were then washed before incubation with a mouse monoclonal antibody specific to human RhoA (1:50 dilution, Santa Cruz Biotechnology) for 1.5 h at room temperature. Samples were washed and incubated with a 1/1000 dilution with 2° antibody (Alexa Fluor 594 goat anti-mouse, Invitrogen) for 1 h at room temperature with gentle agitation. After a final wash step, cells were treated with DAPI as described above.

Coverslips were mounted on to glass microscopic slides using either DAKO Fluorescent mounting medium (Agilent Technologies, California, U.S.A.) or ProLong™ Gold Antifade mountant (Thermo Fisher, Mississauga, ON). Samples were imaged through a 60× oil immersion lens using a Nikon Eclipse Ti-S inverted fluorescence microscope (Nikon Canada; Mississauga, Canada) using the NikonNIS software v4.51. An exposure time of 40 ms was used for DAPI and 100 ms for FITC. Images were analyzed using ImageJ.

## Results

### N-terminal variant production

Seven deletions were made to the N-terminus of C3larvinA. Deletions were made based on the hypothesis that residues of interest would be situated in the N-terminus of C3larvinA before the region that is absent from the truncated C3larvin_trunc_. However, the proposed N-terminal ‘hot-spot’ is expected to be shared with the full-length *P. larvae* toxin, Plx2A—a mART toxin with similar biochemical properties as C3larvinA as described below. For this reason, the first deletion made to the N-terminus of C3larvinA produced a protein of the same length as Plx2A, and the last deletion resulted in a protein of the same length as C3larvin_trunc_.

**Figure 1:**
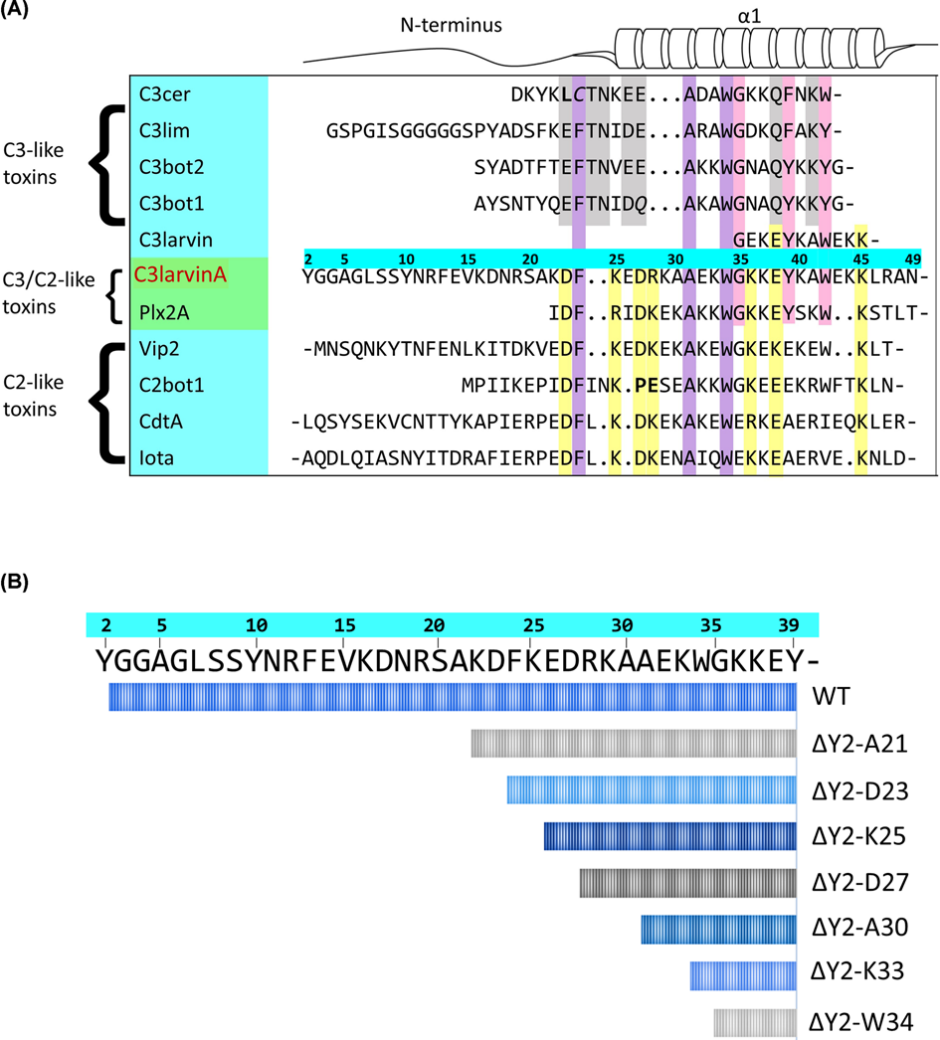
Multiple-sequence alignment of the N-terminus of C3-, C3/C2-, and C2-like toxins (**A**) Residues of the S-motif, conserved in all groups, are shown in purple; residues conserved in C3- and C3/C2-like toxins are shown in pink; residues of the B-motif, conserved in C3/C2- and C2-like toxins, are shown in yellow; residues conserved in C3-like toxins are shown in gray. Bold residues denote deviations from otherwise conserved positions. Gaps within the sequence are denoted by (.) and continuation within a sequence is denoted by (-). Sequence numbers correspond to C3larvinA. (**B**) Graphical depiction of α_1_-helix length in each N-terminal deletion variant. Sequence numbers correspond to C3larvinA.

C3larvinA and Plx2A display C3-like activity, meaning each protein targets RhoA through a catalytic Q-X-E motif, and both can initiate infection in a target cell [28,32]. Despite their shared characteristics with C3-toxins, C3larvinA and Plx2A are unique in that they represent the only proposed binary proteins identified within the subgroup [30–32]. *In silico* analysis revealed that both toxins were encoded by loci containing two genes. In each case, the second gene was identified as a putative B-domain. It is proposed that the respective A- and B-domains associate with one another, as seen in the C2-subgroup of mART toxins, possibly to enhance target cell infection [30–32]. The C2-subgroup of mART toxins function as binary AB toxins, where the catalytic activity is housed in the A-domain and the translocating activity is housed in the B-domain [2,46]. The two proteins are secreted separately, but associate at a target cell membrane through interactions made with the N-terminus of the catalytic A-domain. The B-domain then binds to a membrane-bound receptor to mediate endocytosis [2]. Through a multiple-sequence alignment of CT-toxin N-termini, it was found that Plx2A and C3larvinA share conserved residues with the C2-subgroup (Figure 1A) [42]. This conserved motif was predicted to have a high degree of solvent accessibility and a net charge that could help facilitate electrostatic interactions with another macromolecule. Therefore, these residues were deemed to be a possible site of protein–protein interaction, and were named the Binding-motif (B-motif) [42]. It should be noted, however, that the role of this motif in protein binding is yet to be explored and that the nomenclature used in this paper is meant to be reflective of current literature. The first four residues of the B-motif occurred within the N-terminus of C3larvinA that was not shared with C3larvin_trunc_. As such, these residues were used to select regions for deletion and were also targeted for substitution, resulting in the C3larvinA D23A/K25A/D27A/R28A variant. A second motif was present in the N-terminal extension of C3larvinA that was missing from C3larvin_trunc_, one that was shared among both C2- and C3-toxins. These conserved residues belong to the Structure-motif (S-motif), and were predicted to contribute to protein stability through interactions made with the phospho-nicotinamide (PN)- and ARTT-loops [42]. Again, these residues were used to guide regions for deletion and were targeted for substitution, resulting in the C3larvinA F24A/A31L/W34A variant.

### N-terminal residues structurally support kinetic function

**Figure 2:**
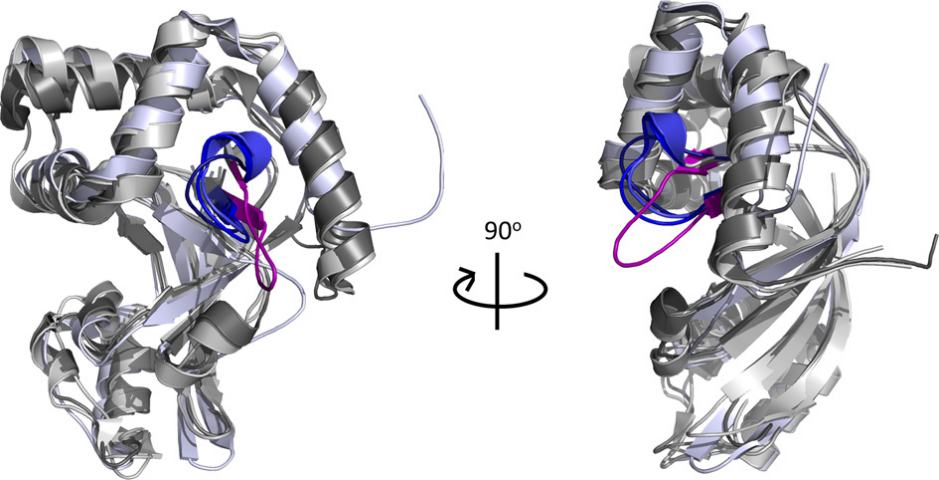
Comparison between the active-site loops of C3larvin_trunc_ and other C3-toxins All toxins are depicted as cartoons. C3larvin_trunc_ (PBD ID: 4TR5, colored light blue) was structurally aligned with C3bot1 (PBD ID: 2C89, colored gray80), C3lim (PBD ID: 3BW8, colored gray40) and Plx2A (PDB ID: 5URP, colored gray70) using PyMOL ver. 1.3. The ARTT-loops of C3bot1, C3lim and Plx2A are colored blue (density, blue and tv_blue, respectively) and the ARTT-loop of C3larvin_trunc_ is colored purple.

The kinetic parameters of each variant were assessed against the NAD^+^ substrate (GH activity) (Table 1). Binding affinity was investigated using β-NAD^+^ as the substrate/ligand and the results showed that variants lacking residue Asp^23^, specifically ΔY2-D23 and D23A/K25A/D27A/R28A (Figure 1B), had lower affinity for the substrate compared with WT (ANOVA, *P*=0.0057) (Table 1). Interestingly, substrate affinity continually improved with subsequent deletions after the removal of Asp^23^, which may suggest a conformational change within the active-site of the enzyme. This is corroborated by the C3larvin_trunc_ crystal structure, which shows an open, more flexible ARTT-loop conformation when compared with other C3-toxins with longer N-termini (Figure 2). The GH activity was then characterized using the fluorescent substrate analog, ε-NAD^+^. The rate of reaction was decreased with each deletion (see Figure 1B for deletion series) until the GH activity reached zero (Table 1). Surprisingly, there was also no detectable reaction for the F24A/A31L/W34A variant; when correlated to the loss of activity of the ΔY2-K33 deletion variant, this result reveals the significance of Ala^31^ in the enzymatic function of C3larvinA. To investigate the role of Asp^23^ and Ala^31^ in substrate-binding and GH activity, respectively, single-residue variants D23A and A31L were further characterized.

**Table 1:** Binding affinity and GH activity of C3LarvinA WT and N-terminal variants against NAD^+^

C3larvinA	*K*_M_ (μM)	*k*_cat_ (min^−1^) × 10^−3^	*k*_cat_/*K*_M_ (M^−1^.min^−1^) × 10^1^	*K*_D_ (μM)
WT	107 ± 20	261 ± 20	244	56 ± 11
ΔY2-A21	120 ± 31	100 ± 10	83	59 ± 2
ΔY2-D23	160 ± 23	46 ± 4	31	117 ± 10
ΔY2-K25	104 ± 27	19 ± 2	18	102 ± 3
ΔY2-D27	112 ± 17	12 ± 0.7	11	93 ± 5
ΔY2-A30	84 ± 17	7 ± 0.4	8	94 ± 3
ΔY2-K33	∼0	∼0	∼0	75 ± 7
ΔY2-W34	∼0	∼0	∼0	38 ± 9
F24A/A31L/W34A	∼0	∼0	∼0	85 ± 14
D23A/K25A/D27A/R28A	28 ± 6	46 ± 2	166	140 ± 14
D23A	94 ± 28	7 ± 0.5	7	131 ± 27
A31L	∼0	∼0	∼0	87 ± 11
K36E	72 ± 12	82 ± 5	114	38 ± 0.4
K36A	75 ± 18	58 ± 4	76	42 ± 2
G35T	∼0	∼0	∼0	113 ± 30
I153A	95 ± 13	40 ± 2	42	85 ± 8
Y178A	99 ± 12	8 ± 0.3	8	65 ± 14

Each value is the average of three replicates ± S.D.; ∼0 = not detectable.

**Figure 3:**
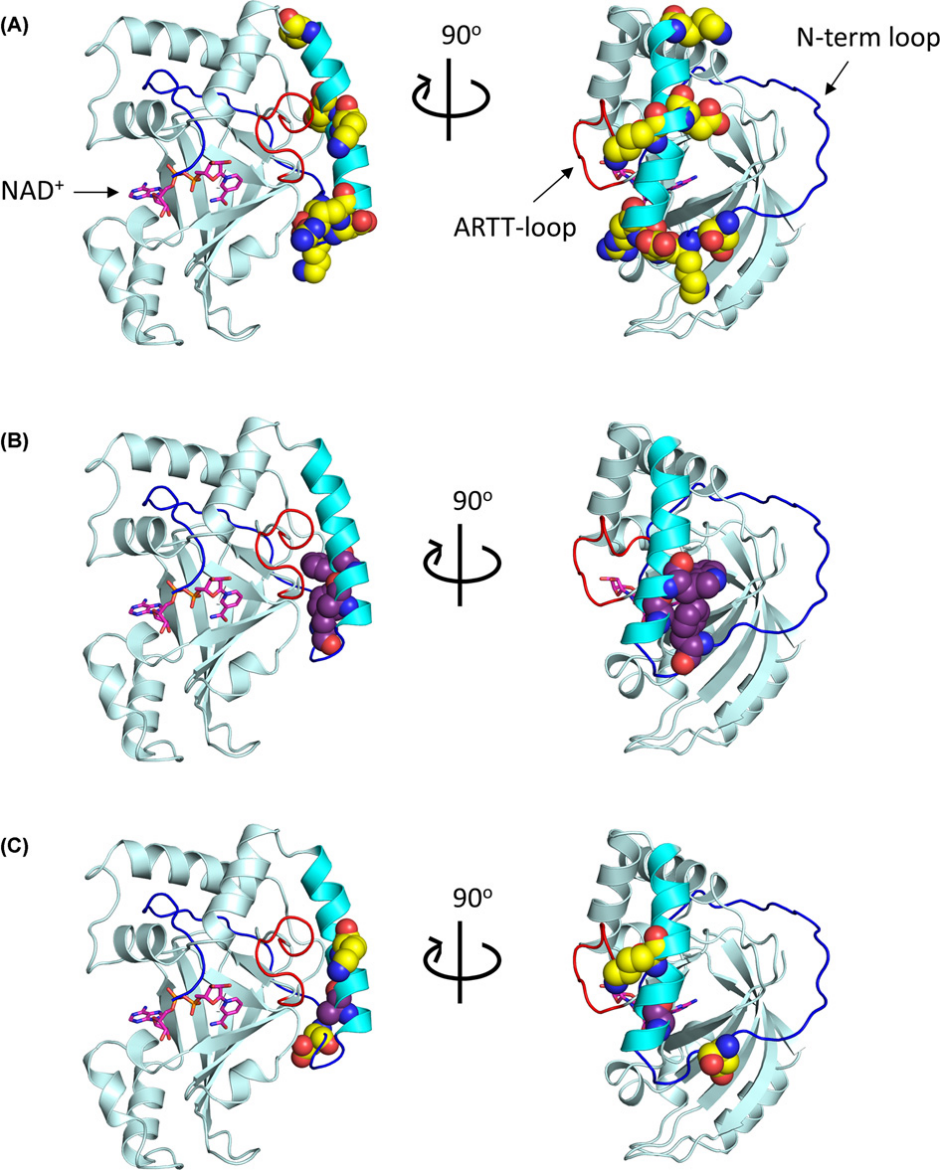
Location of N-terminal motifs in the C3larvinA homology model The C3larvinA homology model is shown in cartoon and colored pale cyan. The ARTT-loop is shown in red; the α_1_-helix is shown in cyan; the unstructured N-terminus is shown in blue. Using the structural alignment tool in PyMOL ver. 1.3, the NAD^+^ substrate (magenta) was modeled into the active site of C3larvinA using the C3bot1–NAD^+^ complex (PDB ID: 2C8F) as a template. (**A**) Residues of the B-motif, D23/K25/D27/R28/K36/E38/K45, are shown using the space-filling model and are colored yellow. (**B**) Residues of the S-motif, F24/A31/W34, are shown using the space-filling model and are colored purple. (**C**) The residues of interest, Asp^23^, Ala^31^ and Lys^36^, are shown using the space-filling model and colored based on their respective motifs (D23 and K36 in yellow; A31 in purple).

The kinetic characterization of D23A and A31L confirmed their role in GH enzymatic function. Substitution of Asp^23^ resulted in a similar *K*_D_ value for NAD^+^ substrate affinity as seen in the ΔY2-D23 and D23A/K25A/D27A/R28A variants, representing a loss of affinity for the NAD^+^ substrate (ANOVA, *P*=0.029). Similarly, GH activity was abolished in the A31L variant, as reported in the ΔY2-K33 and F24A/A31L/W34A variants. Since the residues are members of either the B-motif or S-motif, respectively, it is reasonable to assume that they serve a critical, conserved function (Figure 3). The B-motif, represented by D-(K/R)-D-(K/R)-(K/R)-E-K (Figure 1A, yellow residues), was predicted to mediate the association of the A-domain with its translocating B-domain, or as a possible translocation motif for trafficking the complex into the cytoplasm, for reasons that were previously described [42]. Conversely, the S-motif, which is represented by F-(A/G)-W (FAW motif in C3larvinA) and conserved among both C2- and C3-toxins (Figure 1A, purple residues), was predicted to structurally support the orientation of the α_1_-helix in relation to the ARTT-loop. These N-terminal, nonpolar residues were anticipated to interact with an Ile/Leu within the PN-loop, and a Tyr found on the β_5_-strand was proposed to anchor the protein segments together. A conserved glycine residue from the α_1_-helix also participates, specifically by stabilizing the Tyr^β5^ orientation [42]. To further understand the relationship between kinetic function and the structural location of Asp^23^ and Ala^31^, the C3larvinA homology model was scrutinized and then analyzed.

**Figure 4:**
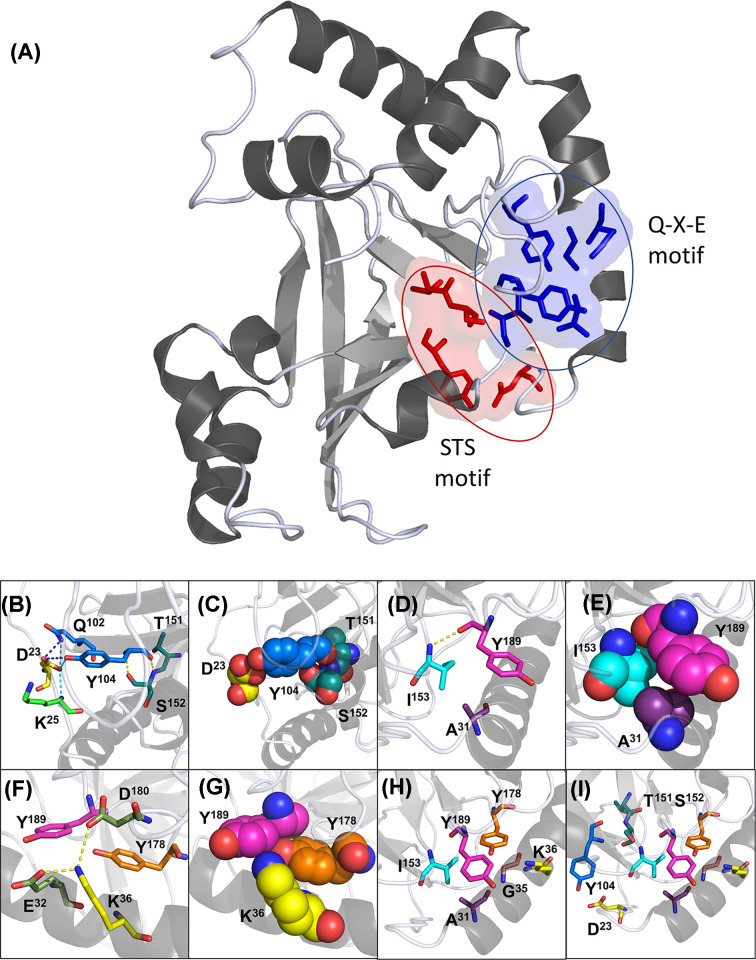
Proposed structural interactions between the N-terminus and catalytic motifs (**A**) The two structural clusters supporting the Q-X-E motif (blue and bound by blue oval) and S-T-S motif (red and bound by red oval). C3larvinA homology model is shown in cartoon, gray70. Residues of interest are shown as sticks and surface (50% transparency), and colored red or blue based on the respective cluster. (**B**–**I**) C3larvinA homology model is shown in cartoon, colored gray70, and set to 60% transparency. All residues are shown as stick or space-filling models and are colored by element. The residues were colored as follows: Asp^23^, yellow; Lys^25^, green; Ala^31^, purple; Glu^32^, smudge; Gly^35^, pink; Lys^36^, yellow; Gln^102^ and Tyr^104^ are marine; Thr^151^ and Ser^152^, teal; Ile^153^, cyan; Tyr^178^, orange; Asp^180^, smudge; and Tyr^189^ is magenta. (**B**) Interactions between Asp^23^ and the S-T-S motif are shown as sticks. Polar interactions formed by the first carboxyl group atom of the Asp^23^ side chain are depicted in cyan. Polar interactions formed by the second carboxyl atom in its side chain are depicted in blue. Polar interactions between Tyr^104^ and Ser^152^ are shown in yellow. (**C**) Space-filling model to depict residue-packing between Asp^23^ and the S-T-S motif. (**D**) Orientation of Ala^31^, Ile^153^ and Tyr^189^ are depicted as sticks. Polar interactions between Ile^153^ and Tyr^189^ are shown in yellow. (**E**) Space-filling model to depict residue-packing between Ala^31^, Ile^153^ and Tyr^189^. (**F**) Orientation of Lys^36^ and Tyr^178^ side chains in relation to Tyr^189^ are shown as sticks. Polar interactions formed by the lysine side chain are shown in yellow. (**G**) Positioning of the Lys^36^ and Tyr^178^ polar side chains toward the phenol ring of Tyr^189^ are depicted in the space-filling model. (**H**) All residues contributing to the orientation of Tyr^189^ are shown as sticks. (**I**) All residues interacting to position both the S-T-S and Q-X-E motifs are shown as sticks.

The C3larvinA homology model built on the shared core with Plx2A (55% identity; PDB: 5URP) revealed that both Asp^23^ and Ala^31^ indirectly affect the orientation of the enzyme catalytic motifs through polar and hydrophobic interactions (Figure 4). Located on the N-terminal loop, the Asp^23^ side chain forms polar interactions with three other residues: Lys^25^, Gln^102^ and Tyr^104^ (Figure 4B). The first oxygen in the carbonyl group of the Asp^23^ side chain interacts with the backbone amino group of Lys^25^, likely as a way of positioning the aspartate side chain toward the center of the protein. The same oxygen then associates with the amino and carbonyl groups of the Gln^102^ and Tyr^104^ side chains, respectively. The other carboxyl oxygen atom of the Asp^23^ side chain also participates in the interaction with Gln^102^ and Tyr^104^, possibly strengthening the resulting orientation of the two residues. It should be noted, however, that this interaction is mediated by a water molecule in the Plx2A crystal structure (data not shown). From this position, the Tyr^104^ backbone carbonyl and amino groups interact with Ser^152^ of the S-T-S motif (Figure 4B,C). The tyrosine amino group hydrogen bonds to the serine hydroxyl side chain, while the carbonyl group forms a likely hydrogen bond with the amino group of the serine backbone. Note that this serine has been previously shown to hydrogen bond with the glutamine of the Q-X-E motif, ensuring proper orientation of the catalytic residue [14].

Within C3larvinA, Ala^31^ faces toward the center of the protein. Herein, it is close to Ile^153^ (identified through computational analysis), as well as to Val^154^, both of which pack against the N-terminus through hydrophobic interactions (Figure 4D,E). The amine-backbone of Ile^153^ forms a polar interaction with the backbone carbonyl group of Tyr^189^, which sits between the catalytic glutamine and glutamate. Substitution of Ala^31^ with a larger leucine would, therefore, force Ile^153^ into an altered conformation that can no longer participate in the positioning of Tyr^189^. Without the proper orientation of the bulky, aromatic tyrosine side chain, the two catalytic residues would become displaced, and no longer positioned for catalysis. It is feasible, then, that other toxins share similar interaction patterns, and that an alanine or glycine is used for this purpose depending on the size constraints of the given toxin.

### Single-residue difference between C3larvin_trunc_ and C3larvinA

A pairwise sequence alignment between C3larvinA and C3larvin_trunc_ revealed a single-residue difference within the α_1_-helix of the two proteins. As a result of a guanine to adenine point mutation, C3larvinA encodes a lysine residue whereas C3larvin_trunc_ contains an oppositely charged glutamate. Notably, this lysine is the fifth residue in the proposed B-motif (Figure 1A), and as the only difference within the shared sequence between C3larvin_trunc_ and C3larvinA, its significance was investigated using the single-residue variants, K36E and K36A.

The kinetic parameters of K36E and K36A against the NAD^+^ substrate was characterized as previously described and are shown in Table 1. These substitutions resulted in a reduced *k*_cat_ for the GH activity when compared with WT, demonstrating a role of Lys^36^ in catalytic function (ANOVA, *P*≤0.0001). Like Asp^23^, Lys^36^ forms a series of interactions serving to position Tyr^189^ in the C3larvinA homology model (Figure 4F). The amino side chain of Lys^36^ was found to form polar interactions with two carboxyl side chains, one from Glu^32^ and one from Asp^180^. These interactions seemingly position the lysine side chain toward the protein center, where it is near Tyr^178^. As stated previously, this tyrosine residue had been identified as a structural support for the N-terminus; however, these interactions had only been predicted to occur with the S-motif, not the B-motif [42]. The 4.1 Å distance between the amino side chain of Lys^36^ and the hydroxyl group of Tyr^189^ suggests the residues may be forming an electrostatic interaction, which is directly oriented toward the phenol ring of Tyr^189^. By directing these polar groups toward the nonpolar benzene ring, Tyr^189^ may be forced into position through repulsion electrostatics. An opposing or lack of charge at this position, therefore, would disrupt these associations and modify the orientation of the Q-X-E motif, accounting for the loss of activity. Discovery of the possible association between Lys^36^ and Tyr^178^ prompted the investigation into the other supportive residues identified *in silico*.

As previously described, there were three residues deemed to associate with the S-motif and contribute to the orientation of the N-terminus toward the body of the enzyme [42]. To further investigate this series of interactions within C3larvinA, the single-residue variants G35T, I153A and Y178A were characterized. Unfortunately, the G35L variant proved to be unstable and was excluded from the present study. In all three variants, the GH activity was compromised (ANOVA, *P*≤0.0001). The largest decrease was seen in the G35T variant, which had no detectable GH activity and showed the greatest increase in *K*_D_ value for the NAD^+^ substrate (ANOVA, *P*=0.048). The GH activity of Y178A, while still being measurable, showed the second largest decrease in *k*_cat_ value with a 33-fold reduction, and I153A yielded the smallest decrease between the three variants, with a 6.5-fold reduction. Both Ile^153^ and Tyr^178^ were previously shown in the present study to be in contact with Tyr^189^, a member of the Q-X-E motif. Therefore, substitution of either residue, or of Gly^35^, likely results in altered residue-packing, shifting the orientation of the catalytic motif and affecting the enzymatic activity of the protein. These findings reinforce the designation of these residues as being structurally supportive to the enzyme active-site.

### Stability of single-residue variants

Circular dichroism (CD) spectroscopy was used to probe for changes in secondary structure caused by residue substitutions. All variant CD spectra were comparable with that of WT and it was concluded that the substitutions did not have a significant effect on protein structure (Supplementary Figure S1). The bioinformatics tool, STRUM, and differential scanning fluorimetry (DSF) were then used to assess the impact of each single-residue substitution on protein stability. STRUM analysis predicts the change in protein folded stability, while DSF directly measures the change in protein thermal stability. All residues identified through computational studies as structurally supportive, Ala^31^, Gly^35^, Ile^153^, and Tyr^178^, were predicted to contribute to protein folded stability (Table 2). This means that, in the STRUM analysis, substitution of these residues resulted in a negative ΔΔG score, indicating a loss of stability. The two residues predicted to have the greatest impact on protein stability were Ile^153^ and Tyr^178^. Alanine substitution of either residue resulted in a ΔΔG score near −2, and correspondingly, the T_M_ values of each variant were reduced by 5 and 4°C, respectively, when compared with WT. The substitutions A31L and G35T were predicted to be mildly destabilizing, with both having a ΔΔG score approximately −0.4. Interestingly, through DSF analysis, both variants displayed significant decreases in T_M_ values, like those seen in the I153A and Y178A variants, with a 4 and 3°C reduction, respectively. At last, both Asp^23^ and Lys^36^ were predicted to be destabilizing to protein folded stability. The alanine-substitution of Asp^23^ resulted in a ΔΔG score of +0.16, however, DSF analysis for D23A showed a 3°C decrease in T_M_ value. Conversely, the Lys^36^ variants, K36E and K36A, yielded high ΔΔG scores of +0.65 and +0.83, respectively, and both showed increases in thermal stability. The K36E variant had a 3.6°C increase in T_M_ value, the largest seen in any variant, while K36A had a modest 0.6°C increase.

**Table 2:** Thermal stability of single-residue variants determined by DSF and predicted fold-stability of each substitution by STRUM analysis

Protein	T_m_ (°C)^1^	ΔT_m_ (°C)^2^	ΔΔG_calc (kcal/mol)_^3^
I153A	57.3 ± 0.1	−5	−1.98
Y178A	58.4 ± 0.1	−4.1	−1.79
A31L	58.4 ± 0.1	−4.1	−0.47
G35T	59.4 ± 0.1	−3.1	−0.45
D23A	59.8 ± 0.1	−2.7	+0.16
K36A	63.1 ± 0.1	+0.6	+0.83
K36E	66.1 ± 0.1	+3.6	+0.65
WT	62.5 ± 0.1	N/A	N/A

1 The T_m_ values were determined from the thermal melt curves for each C3larvinA protein using Thermal Shift Software v1 (Applied Biosystems).

2 The ΔT_m_ values were calculated from the difference between the WT T_m_ (62.5°C) and each variant (Δ T_m_ **_=_** T_m variant_ − T_m wild-type_).

3 The ΔΔG values (kcal/mol) for folded stability change for each substitution variant were calculated using the STRUM algorithm where ΔΔG = ΔG _wild-type_ − ΔG _variant_. A ΔΔG value above zero indicates the substitution is stabilizing, while a value under zero indicates the substitution is destabilizing.

### Cell entry experiments

**Figure 5:**
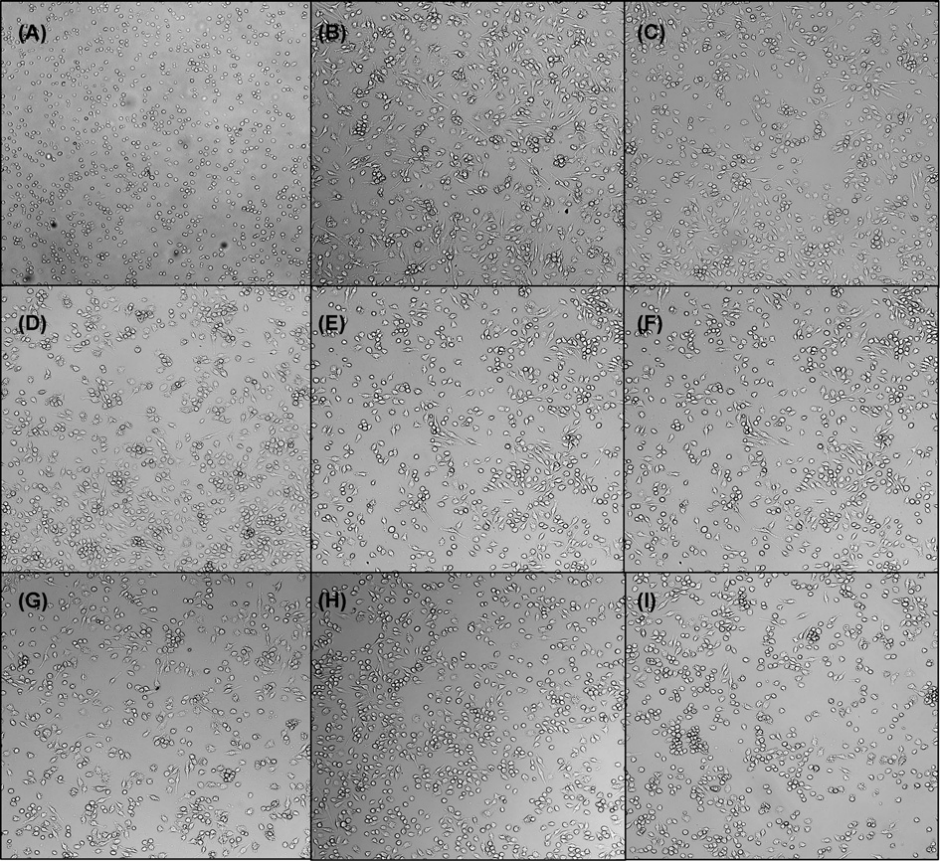
C3larvinA N-terminal deletions have varying effects on the morphology of macrophage cells J774A.1 mouse macrophage cells were treated with 300 nM toxin and incubated for 20 h at a cell density of 37500 cells/well. Effects to morphology were less dramatic in the larger N-terminal deletions. (**A**) Buffer control; (**B**) C3larvinA (full length); (**C**) ΔY2-A21; (**D**) ΔY2-D23; (**E**) ΔY2-K25; (**F**) ΔY2-D27; (**G**) ΔY2-A30; (**H**) ΔY2-K33; (**I**) ΔY2-W34.

The seven deletions shown in Figure 1B were tested against J774A.1 murine macrophage cells at a final concentration of 300 nM. After a 20-h incubation period, cells were assessed for morphological changes associated with C3-toxin infection (Figure 5). As previously demonstrated for C3larvinA, the WT protein at 300 nM (Figure 5B) showed morphology changes indicating host cell entry and disruption of the RhoA function, the physiological target of the toxin [29]. Morphological changes caused by C3-toxin treatment are characterized by enlarged cells with filopodia-like protrusions, with the most obvious changes occurring in the J774A.1 murine macrophage cell line [47]. Interestingly, the extent of the morphology changes seen in the variant-treated cells appeared to be inversely related to the length of the protein (Figure 5C–I). However, it was unclear whether these findings were based on the ability of each toxin variant to penetrate the cell membrane, or due to weakened catalytic activity of the shorter variants. Fluorescence microscopy experiments were then developed as a means of differentiating between cell entry and enzymatic function.

**Figure 6:**
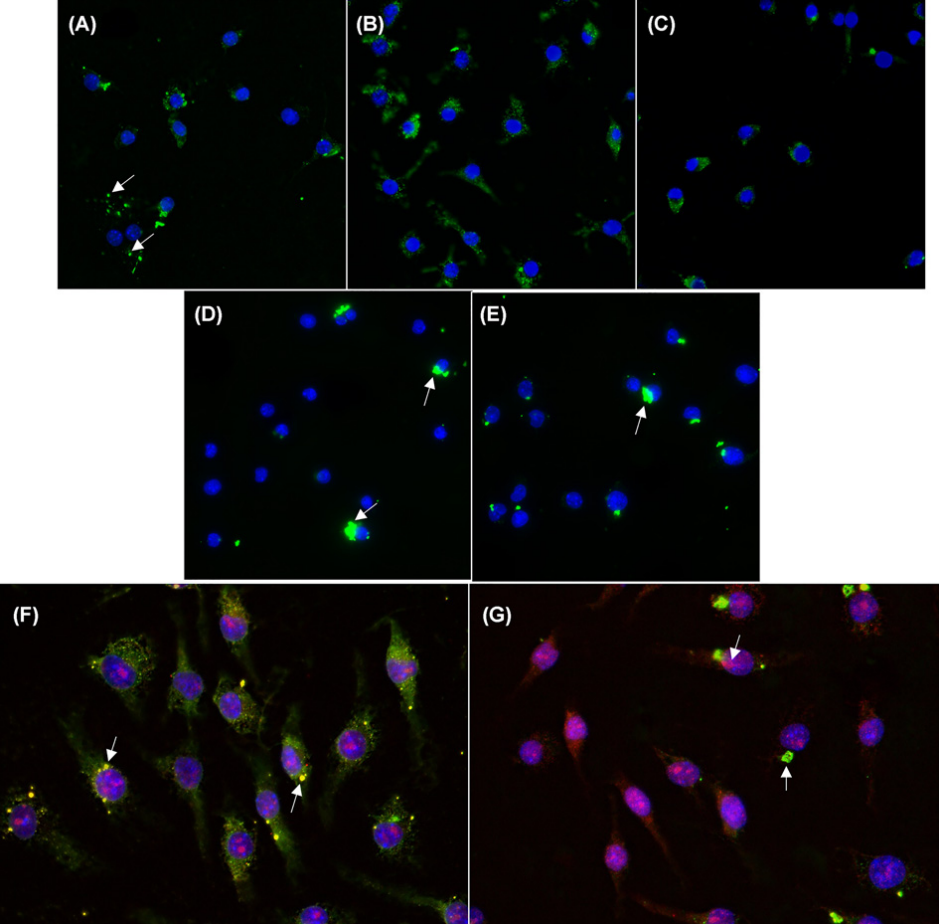
Fluorescence microscopy of J774A.1 mouse macrophage cells treated with toxins conjugated with Dylight 488 Macrophage cells were seeded overnight at 500000 cells/well before subsequent treatment with 300 nM of toxin for 4 h. Samples were exposed for 40 ms at 358 nm for DAPI and 100 ms at 495 for FITC. Data were analyzed using ImageJ. (**A**) C3larvinA WT; (**B**) C3larvinA ΔY2-A31; (**C**) C3larvinA ΔY2-W34; (**D**) C3larvin_trunc_; (**E**) C3larvinA ΔY2-W34 K36E. **Co-localization of C3larvinA and C3larvin_trunc_ with cellular RhoA.** Mouse macrophage J774A.1 cells were seeded overnight at 250000 cells/ml before treatment with 300 nM of toxin conjugated with Dylight 488 for 4 h. Samples were exposed for 40 ms at 358 nm for DAPI, 100 ms at 495 nm for FITC, and 100 ms at 532 nm for TRITC. Data were analyzed using ImageJ. (**F**) C3larvinA-treated cells and (**G**) C3larvin_trunc_-treated cells.

Purified toxin was conjugated to an amine-reactive, green fluorophore to allow for visualization in the cell experiments. The toxin–conjugate was then incubated with macrophage cells for 4 h at a final concentration of 300 nM. Afterward, cells were thoroughly washed, fixed and treated with DAPI to stain the nucleus. WT C3larvin_trunc_ and C3larvinA toxins were used as controls and acted as references to classify the phenotypes arising from the different variants tested. The C3larvinA phenotype showed the appearance of small, dispersed clusters (Figure 6A, arrows). Conversely, C3larvin_trunc_-treated cells exhibited a phenotype in which the protein appeared as larger puncta within the cell (Figure 6D, arrows). This phenotype was clearly distinctive from that of C3larvinA (Figure 6A). Two N-terminal deletions, C3larvinA ΔY2-A31 and C3larvinA ΔY2-W34 (Figure 6B,C) displayed phenotypes like that of C3larvinA. Attention was then shifted to the single-residue difference between ΔY2-W34 and C3larvin_trunc_, resulting in the addition of the ΔY2-W34 K36E variant to the present study. Figure 6E (see arrow) reveals that this variant had an identical protein sequence and similar cellular phenotype to C3larvin_trunc_, suggesting a role of Lys^36^ and/or net charge in the translocation activity of the enzyme.

A final study was conducted to further investigate the different cellular phenotypes displayed in C3larvin_trunc_- and C3larvinA-treated macrophage cells. When combining the current results from those of previous studies, namely that C3larvin_trunc_ fails to elucidate morphology changes within treated cells, it is reasonable to assume that the toxin is failing to target cellular RhoA, despite its ability to modify the G-protein *in vitro* [32]. It is also important to note that, in a yeast-based assay, C3larvin_trunc_ showed strong cytotoxicity against yeast cells when the toxin was expressed in the cytoplasm under the control of a *CUP1* promoter [28,32]. This yeast cell cytotoxicity was dependent on the enzymatic activity of the toxin since catalytically inactive variants showed no cell killing effects [48]. These studies suggest that C3larvin_trunc_ is catalytically capable of disrupting cellular function in macrophage cells yet is unable to do so when added into the extracellular medium. In contrast, C3larvinA causes extensive morphology changes in toxin-treated macrophage cells, indicating it is successfully modifying its target substrate [29]. This theory was tested through a co-localization study in which cellular RhoA was targeted using antibodies conjugated to Alexa Fluor 595, a red fluorophore. As expected, the fluorescence from both C3larvinA and RhoA overlapped, resulting in a yellow colour, indicating colocalization of the toxin and its RhoA target (Figure 6F, arrows). Conversely, samples treated with C3larvin_trunc_ largely appeared green (C3larvinA) and red (RhoA) (Figure 6G, arrows), indicating that the RhoA protein was not being targeted by the green C3larvin_trunc_ toxin–conjugate. Therefore, despite being visible within the cell, the C3larvin_trunc_ toxin fails to target/modify cellular RhoA in live mammalian cells.

## Discussion

N-terminal residues within the α_1_-helix of the C3larvinA toxin from *P. larvae* were probed to determine their functional role in cell entry and RhoA modification. The extended N-terminus of C3larvinA imparts the ability to target cellular RhoA while significantly improving its catalytic ability when compared with the truncated, C3larvin_trunc_ [28,32]. Deletions and point-mutations within the extended N-terminal region revealed key structural interactions between the α_1_-helix and the active-site of the enzyme. Residues previously identified through computational studies were shown to participate in the orientation of the N-terminus and demonstrated their important role in the enzymatic activity of the protein. Additionally, novel interactions were discovered, revealing the structural role of two additional residues important to catalytic function. Interestingly, only a single residue was found to participate in the cell translocation function of the toxin.

Deletions were made to the C3larvinA N-terminus based on a 13-residue region of interest. The consistent decrease in *k*_cat_ values with subsequent deletions shows the importance of N-terminal interactions to manage enzymatic function. Removal of these interactions likely allows more flexibility in the ARTT-loop, resulting in conformations that are catalytically less efficient. Evidence of this can be seen in the C3larvin_trunc_ crystal structure, which has an extended ARTT-loop conformation when compared with other C3-toxin structures. The increased flexibility may allow the substrate-binding site to adopt additional protein conformations, some of which may not be catalytically active (or less so). While not as efficient for enzymatic function, the open conformation has similar affinity for the NAD^+^ substrate. The loss of interactions of the enzyme core with the N-terminus may allow the protein to increase interactions with the NAD^+^ molecule and form a tighter but less catalytically efficient Michaelis complex. Characterization of these variants along with the two multiresidue variants, D23A/K25A/D27A/R28A and F24A/A31L/W34A, led to the discovery that Asp^23^ and Ala^31^ play important secondary roles in substrate-binding and catalytic activity.

A network of interactions between the N-terminus and key catalytic residues was uncovered through the investigation of Asp^23^ and Ala^31^, and later through the investigation of Lys^36^. The interactions can be classified into two main clusters, which orient either the Q-X-E or S-T-S motifs. Within the first cluster, the semi-conserved alanine residue from the S-motif, and lysine residue from the B-motif contribute to the orientation of Tyr^189^. Residue packing between the Ala^31^ and Ile^153^ side chains directs the peptide bond of Ile^153^ to interact with the corresponding bond in Tyr^189^. The Tyr^189^ side chain is then oriented through repulsion electrostatics via the combined positions of the Lys^36^ and Tyr^178^ side chains (Figure 4A). The B-motif is also responsible for the second cluster of interactions which orient the S-T-S motif (Figure 4H). The Asp^23^ side chain positions the peptide bond of Tyr^104^ allowing for the formation of hydrogen bonds with Ser^152^. Interestingly, these two clusters of interactions are connected through the hydrogen bonds formed between the S-T-S and Q-X-E motifs (Figure 4I), which contribute to the structure of the active-site [10,14].

The contribution of each residue to protein stability was assessed to better understand their respective roles in enzyme structure and function. The substitution of both Ile^153^ and Tyr^178^ were predicted to be largely destabilizing, and indeed the respective T_M_ values supported this prediction. These findings further substantiated the classification of these residues as structural, and the loss of activity associated with their substitution indicates that this interaction is not only beneficial to protein stability, but also to enzymatic function. Interestingly, G35T and A31L were predicted to be mildly destabilizing; however, the T_M_ values were reduced by at least 3°C, like those of I153A and Y178A. All four residues were identified *in silico* as participating in the interaction between the α_1_-helix and ARTT-loop [2]; therefore, substitution would be expected to alter residue packing between these structures. This could lead to increased flexibility within the protein, causing the decrease in thermal stability and producing more protein conformations with an overall reduction in catalytic efficiency reflected in the reduced GH activity. At last, the substitution of either Asp^23^ or Lys^36^ were both predicted to be stabilizing to protein structure, but only the substitution of Lys^36^ showed this effect. Since Lys^36^ and Tyr^178^ work to position Tyr^189^ through repulsive forces, the replacement of this residue would be expected to have a stabilizing effect on protein structure. However, this interaction is also key to proper enzymatic function in ensuring the orientation of the Q-X-E motif into a catalytically efficient conformation, hence, the Lys^36^ substitution caused a loss of GH activity.

In addition to enzymatic function and protein stability, residues within the N-terminus of C3larvinA were predicted to contribute to cell intoxication. This prediction was based on the morphological changes seen in C3larvinA-treated macrophage cells, and the lack of changes in cells treated with C3larvin_trunc_ [29,32]. As previously stated, these changes in morphology represent a loss of activity of cellular RhoA, indicating that the toxin has successfully infected the target cell and associated with its physiological target. It was previously shown that a 17-residue N-terminal extension from the α_1_-helix of C3bot1 on to C3larvin_trunc_ allowed C3larvin_trunc_ to elicit morphology changes within macrophage cells, similar to full-length C3 toxins [32]. This suggested that critical residues responsible for mediating cell entry are in the N-terminus. To date, very little is known about the cellular uptake of C3 toxins. Some research has suggested that C3 toxins are internalized into endosomes and traverse into the cytoplasm during the acidification process; others propose that C3 toxins bind membrane-bound vimentin using an RGD motif to gain access to the cell [47,49]. Currently, the only widely accepted theory is that these toxins are selectively internalized and inevitably locate to cytoplasmic Rho-GTPases.

To investigate the membrane-translocation activity of the N-terminal variants, a fluorescence microscopy assay was developed. Interestingly, C3larvin_trunc_, which was previously shown to be unable to enter cells, was visible inside macrophages in fluorescence microscopy experiments [32]. Therefore, while the protein may be unable to exert toxic effects on the cell, these findings suggest that the truncated toxin can gain access to the host cell by translocating across the plasma cell membrane. Co-localization studies confirmed that C3larvin_trunc_ was not targeting the RhoA substrate, leading to the possibility that, although the protein can enter host cells, it may be trapped within the vesicle system that parades from the plasma membrane to the cytoplasm. Conversely, C3larvinA displayed a dispersed phenotype in the cell and was shown to colocalize with RhoA. A similar phenotype was seen in all N-terminal deletions that were tested, including the ΔY2-W34 deletion, which is the same length as C3larvin_trunc_. Importantly, there is a single-residue difference found at position 36 between C3larvinA ΔY2-W34 and C3larvin_trunc_ proteins that may account for the different phenotypes: Lys compared with Glu in C3larvinA and C3larvin_trunc_, respectively. A final variant, ΔY2-W34 K36E, was therefore tested in the assay and confirmed to share a phenotype with C3larvin_trunc_.

As a member of the B-motif, Lys^36^ is not conserved among C3-toxins, raising the question of how it contributes to cell entry. If C3larvinA shares a mode of entry and translocation like that of other C3-toxins, the importance of this residue may lie solely in its charge. The inability of C3larvin_trunc_ to exert toxic effects on target cells without an N-terminal addition from C3bot1 led to the original interpretation that C3larvin_trunc_ was lacking the machinery required to gain access to the cell. Given the current findings, however, it is possible that the residues from the N-terminal additional segment imparted C3larvin_trunc_ with the ability to escape the vesicle system and translocate into the cell cytoplasm. This change may not have been due to the specific residues or a given length but may instead be based on the protein net or global charge. Computational analysis of C3larvin_trunc_ with and without the N-terminal addition revealed that there was a shift in protein dipole when the N-terminus was extended [42]. This change also made the dipole more comparable with that of other C3-toxins, possibly indicating the importance of this global charge to cell intoxication, as seen previously [42]. Due to the largely unknown nature of the cellular pathway taken by C3-toxins, it is difficult to say how this charge is affecting translocation, and this observation will require further studies.

C3larvinA offers many unique opportunities for future research. It shares qualities between both the C2- and C3-subgroups that may prove insightful to the evolutionary relationship between the two groups. Namely, identifying equivalent residues in C3-toxins to the B-motif, which has been shown in this work to be supportive to enzymatic function in C3larvinA, is an interesting avenue for research pursuit. Furthermore, the possible relationship between protein dipole and cellular localization may prove useful to extending the current understanding of cellular intoxication, and therefore, warrants additional examination.

## Supplementary Material

Supplementary Figure S1

## Data Availability

All data shown in this work are readily available upon request from the authors at rmerrill@uoguelph.ca. The C3larvinA homology model was built with Phyre2 http://www.sbg.bio.ic.ac.uk/∼phyre2/html/page.cgi?id=index using the PDB: ID 5URP, found at https://www.rcsb.org/.

## Abbreviations

AFB: American Foulbrood
ARTT: ADP-ribosyl-turn-turn
CD: circular dichroism
DAPI: 2-(4-amidinophenyl)-1H-indole-6-carboxamidine
ERIC: enterobacterial repetitive intergenic consensus
GH: glycohydrolase
mART: mono-ADP-ribosyltransferase
PN: phospho-nicotinamide
WT: wild-type
ɛ-AMP: etheno-adenosine monophosphate
ɛ-NAD^+^: etheno-β-nicotinamide adenine dinucleotide
β-NAD^+^: β-nicotinamide adenine dinucleotide
