# Correction: The N-terminus of *Paenibacillus larvae* C3larvinA modulates catalytic efficiency

**DOI:** 10.1042/BSR-20203727_COR

**Published:** 2021-04-27

**Keywords:** ADP-ribosyltransferase toxins, enzyme mechanisms, honey bee diseases, macrophage cell entry, protein-protein interactions

The authors of the original article “The N-terminus of *Paenibacillus larvae* C3larvinA modulates catalytic efficiency” (*Biosci Rep* (2021) 41(1), **DOI**: 10.1042/BSR20203727) would like to correct Figure 5 of their article. During figure preparation for the submission of their manuscript, there had been an inadvertent duplication of Panel E which resulted in the replacement of Panel F in Figure 5. The authors clarify that Panels E and F were different variants of the C3larvinA toxin that produced identical phenotypes when infecting host macrophages. They further state that this duplication of panel E in Figure 5 does not change the conclusion of the results in Figure 5 or in the paper. The correct Figure 5 is present in this Correction. The authors apologise for any inconvenience caused by this error.

**Figure 5:**
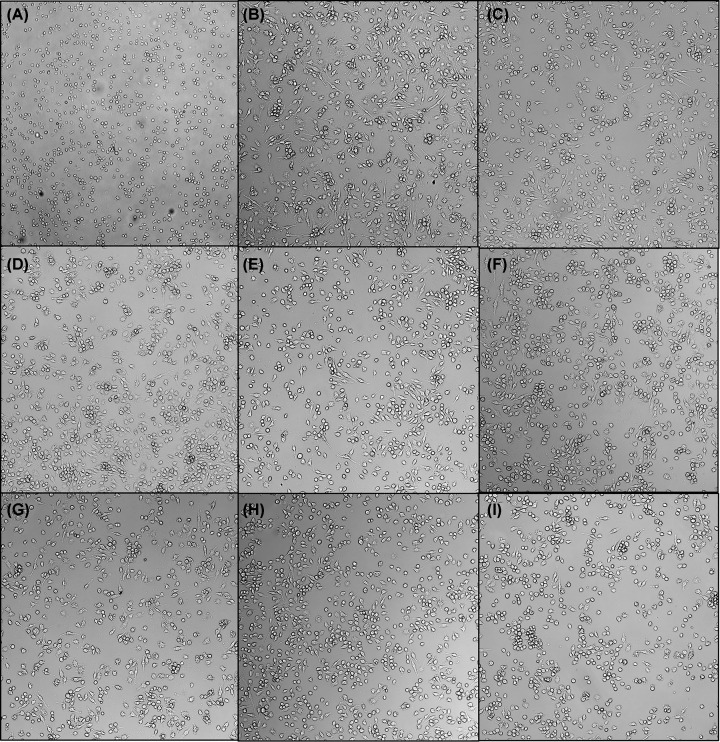
C3larvinA N-terminal deletions have varying effects on the morphology of macrophage cells J774A.1 mouse macrophage cells were treated with 300 nM toxin and incubated for 20 h at a cell density of 37500 cells/well. Effects to morphology were less dramatic in the larger N-terminal deletions. (**A**) Buffer control; (**B**) C3larvinA (full length); (**C**) ΔY2-A21; (**D**) ΔY2-D23; (**E**) ΔY2-K25; (**F**) ΔY2-D27; (**G**) ΔY2-A30; (**H**) ΔY2-K33; (**I**) ΔY2-W34.

