# Supplementary material for: The N-terminus of Paenibacillus larvae C3larvinA modulates catalytic efficiency
Source: Biosci Rep. 2021 Jan 6;41(1):BSR20203727. doi: 10.1042/BSR20203727 (PMC7789906; doi:10.1042/BSR20203727)
Supplement: Supplementary Figure S1 [file BSR-2020-3727_supp.pdf]

Supplemental Information

Supplemental Figure S1. *CD spectra of C3larvinA WT and variants.* (A) WT, black; ΔY2-A21, red; ΔY2-D23, med blue; ΔY2-K25, green; ΔY2-D27, magenta; ΔY2-A30, orange; ΔY2-K33, navy blue; ΔY2-W34, wine. (B) WT, black; D23A, magenta; A31L, gray; I153A, red; Y178A, mid blue; F24A/A31L/W34A, orange; D23A/K25A/D27A/R28A, olive. (C) WT, black; ΔW34K2A, red; K36E, mid blue; K36A, magenta. Purified C3larvinA WT and catalytic variants were dialyzed into buffer containing 250 mM NaF and 10 mM Tris-HCl, pH 7.5. A JASCO J-815 CD spectropolarimeter (JASCO-USA, Silver Spring, USA) was used to acquire the circularly polarized spectra of C3larvinA WT and variant proteins (0.16 mg/mL) at 25°C in a 1 mm pathlength cuvette by scanning from 250 – 190 nm for a total of 9 scans from which an average spectrum was calculated.

A.

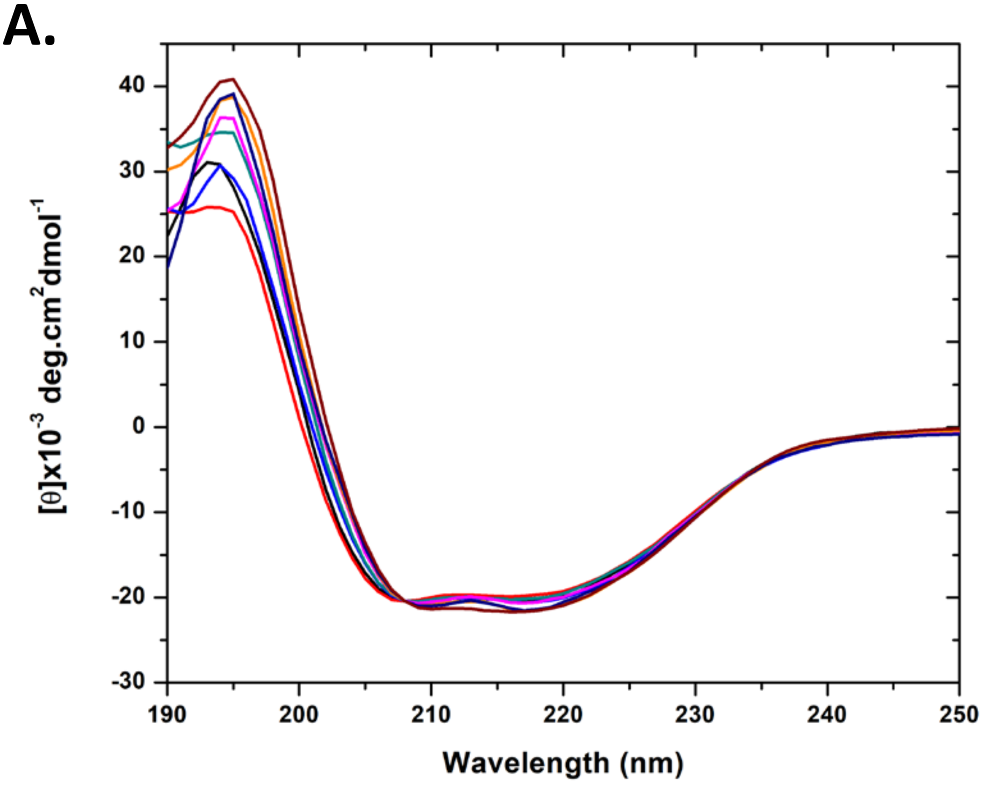

B.

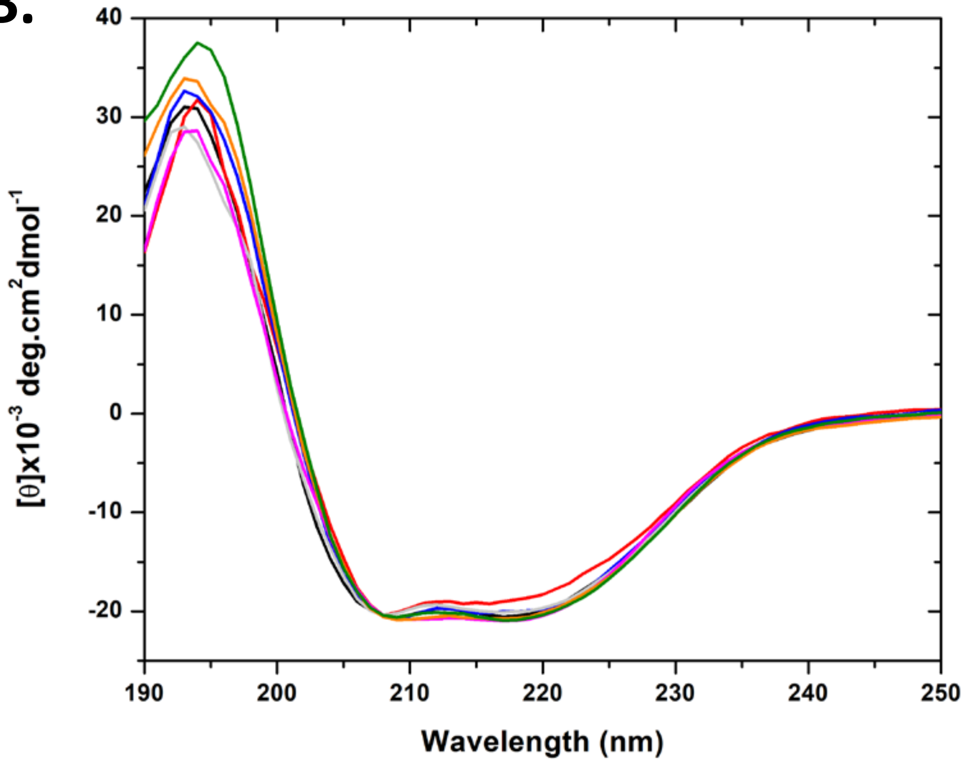

40
30
20
10
0
-10
-20
190
200
210
220
230
240
250
[θ]x10^{-3} deg.cm^{2}dmol^{-1}
Wavelength (nm)

C.

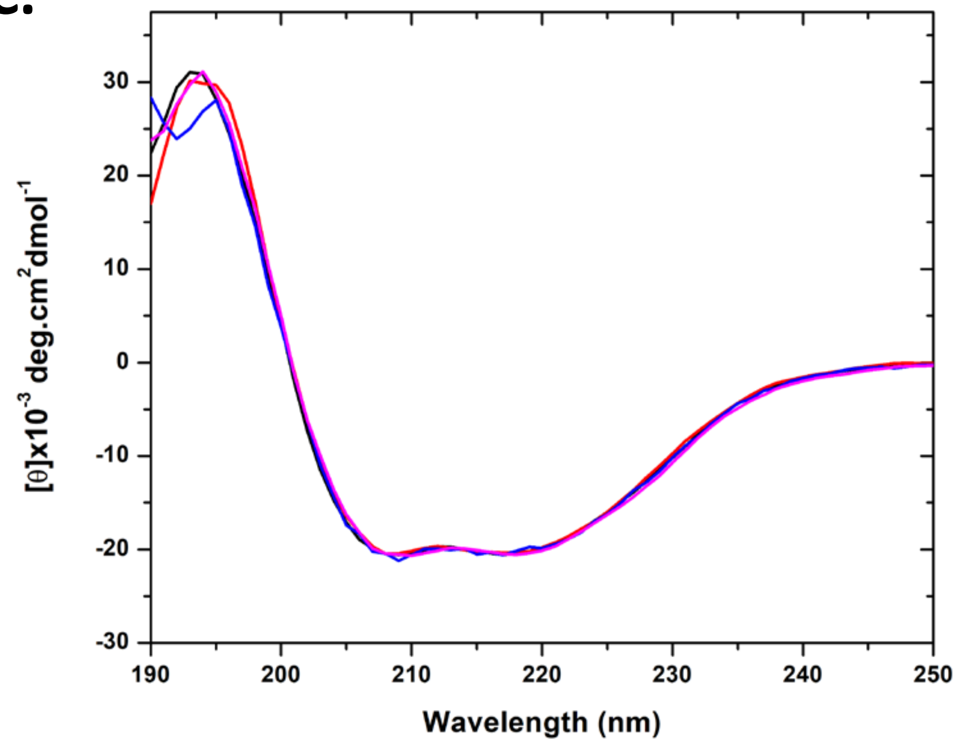

30
20
10
0
-10
-20
-30
190
200
210
220
230
240
250
[θ]x10^{-3} deg.cm^{2}dmol^{-1}
Wavelength (nm)
